# Treatment With the Delta Opioid Agonist UFP-512 Alleviates Chronic Inflammatory and Neuropathic Pain: Mechanisms Implicated

**DOI:** 10.3389/fphar.2019.00283

**Published:** 2019-03-26

**Authors:** Sara Polo, Andrés Felipe Díaz, Núria Gallardo, Sergi Leánez, Gianfranco Balboni, Olga Pol

**Affiliations:** ^1^Grup de Neurofarmacologia Molecular, Institut d’Investigació Biomèdica Sant Pau, Barcelona, Spain; ^2^Grup de Neurofarmacologia Molecular, Institut de Neurociències, Universitat Autònoma de Barcelona, Barcelona, Spain; ^3^Department of Life and Environmental Sciences, Unit of Pharmaceutical, Pharmacological and Nutraceutical Sciences, University of Cagliari, Cagliari, Italy

**Keywords:** analgesia, depression, Nrf2 transcription factor, delta opioid receptors, oxidative stress, chronic pain, UFP-512

## Abstract

We investigated whether administration of the δ-opioid receptor (DOR) agonist H-Dmt-Tic-NH-CH(CH2-COOH)-Bid (UFP-512), which also activates nuclear factor erythroid 2-related factor 2 (Nrf2), alleviated chronic inflammatory and/or neuropathic pain and inhibited the depressive-like behaviors associated with persistent neuropathic pain. The possible mechanisms implicated were also assessed. We evaluated the following effects in male C57BL/6J mice with inflammatory pain induced by *complete Freund’s adjuvant* or neuropathic pain caused by the chronic constriction of sciatic nerve: (1) the antinociceptive effects of UFP-512; (2) the effects of UFP-512 on the expression of Nrf2, heme oxygenase 1 (HO-1), NAD(P)H quinone oxidoreductase 1, phosphoinositide 3-kinase (PI3K), protein kinase B (Akt), inducible nitric oxide synthase, DOR, and mitogen-activated protein kinases (MAPK) in the spinal cord of animals with inflammatory or neuropathic pain; (3) the antinociceptive effects of the coadministration of UFP-512 with the Nrf2 activator sulforaphane (SFN); and (4) the antidepressant effects of UFP-512 in animals with depressive-like behaviors associated with neuropathic pain. Our results demonstrated that the intraperitoneal administration of UFP-512 inhibited chronic inflammatory and neuropathic pain and reduced the depressive-like behaviors associated with persistent neuropathic pain. The antiallodynic effects of UFP-512 were significantly augmented when it was coadministered with SFN in both types of chronic pain. The administration of UFP-512 increased/reestablished the spinal cord protein levels of Nrf2 and HO-1 in mice with inflammatory or neuropathic pain. However, while during inflammatory pain UFP-512 inhibited spinal c-Jun N-terminal kinase (JNK) and extracellular signal regulated kinase 1/2 (ERK1/2) phosphorylation induced by peripheral inflammation. This DOR agonist blocked the spinal activated PI3K/Akt signaling pathway under chronic neuropathic pain conditions, but it did not alter the enhanced protein levels of p-JNK or p-ERK1/2 induced by sciatic nerve injury. These results revealed the antinociceptive and antidepressant effects of UFP-512 in animals with chronic pain and the different mechanism of action of this DOR agonist in the presence of inflammatory or neuropathic pain. Our data also suggest the administration of UFP-512 as an alternative for the treatment of chronic pain and the depressive-like behaviors associated with neuropathic pain.

## Introduction

Chronic pain is a complex experience that is composed of sensory and affective components. Therefore, when pain becomes persistent or severe causes great suffering that presents an important clinical problem with enormous economic and social burdens (Monti and Caporali, 2015). One factor that contributes to the intractable nature of chronic pain is the existence of comorbidities associated such as depression, which also negatively influence the perception of pain and create a vicious circle that eventually leads to unwanted results (Kawai et al., 2017).

Chronic inflammatory and neuropathic pains are difficult to treat because actual therapies, such as NSAIDs, antidepressants, and antiepileptics, have several side effects, and these agents do not effectively alleviate pain and/or the associated emotional disorders. The opioid system plays a crucial role in pain control, and activation of μ-(MOR), δ-(DOR), and κ-opioid receptors produces analgesia (Pol, 2007; Cahill et al., 2014; Albert-Vartanian et al., 2016; Bodnar, 2016). However, it is curious that while the administration of MOR agonists is highly effective for the treatment of inflammatory pain, these agents exhibit limited efficacy in neuropathic pain and produce several adverse side effects, such as sedation, constipation, and respiratory depression. In contrast, and although the administration of several DOR agonists produce limited analgesic effects in acute pain, these agonists reduced chronic inflammatory and neuropathic pain with similar efficacy (Pradhan et al., 2011). Moreover, DOR knockout (KO) mice exhibited increased inflammatory and neuropathic pain responses when submitted to an inflammatory or injury stimulus (Nadal et al., 2006), which supports the hypothesis of DOR agonists as an interesting alternative for the treatment of chronic inflammatory and neuropathic pain. However, comparisons of the antinociceptive effects of several DOR agonists, such as [D-Ser2, Leu5, Thr6]-enkephalin (DSLET) and (+)-4-[(αR)-α-((2S,5R)-4-Allyl-2,5-dimethyl-1-piperazinyl)-3-methoxybenzyl]-*N*,*N*-diethylbenzamide (SNC-80), in inflammatory and neuropathic pain were only performed in models of acute inflammatory pain (formalin test) or in the early stages of neuropathic pain (16 days after surgery) (Obara et al., 2009). Therefore, to the present study we evaluated the antinociceptive effects produced by another specific DOR agonist, H-Dmt-Tic-NH-CH(CH2-COOH)-Bid (UFP-512), in animals with chronic inflammatory (14 days after induction) or neuropathic (28 days after surgery) pain. We used UFP-512 because this agonist did not produce convulsions or alter locomotor activity, and it exhibits anxiolytic and antidepressant properties without signs of tolerance after chronic administration in contrast to other DOR agonists, for example, UFP-502 (Vergura et al., 2006, 2008; Aguila et al., 2007).

Several studies also showed the potential use of some DOR agonists, such as [D-Pen^2^,D-Pen^5^]-enkephalin (DPDPE), SNC-80, and UFP-512, as antidepressant drugs (Vergura et al., 2008; Lutz and Kieffer, 2013), and these effects were also confirmed by an increase in depressive-like behaviors in DOR-KO mice in different depressive animal models (Filliol et al., 2000). It is also well known that persistent neuropathic pain is associated with several comorbidities, such as depression, which is difficult to treat with typical antidepressants (La Porta et al., 2016). Notably, we recently demonstrated that treatment with the nuclear factor erythroid 2-related factor 2 (Nrf2) inducer sulforaphane (SFN) inhibited the depressive-like behaviors linked with chronic neuropathic pain (Ferreira-Chamorro et al., 2018). *In vitro* studies also revealed that UFP-512 induced activation of Nrf2 (Cao S. et al., 2015). Therefore, we evaluated the possible antidepressant effects of UFP-512 in animals with depressive-like behaviors associated with persistent neuropathic pain.

The contribution of oxidative stress to central and peripheral sensitization (Meeus et al., 2013; Riego et al., 2018) and the protective role played by Nrf2 in reducing oxidative stress via enhancing the expression of several antioxidant or detoxification enzymes, including heme-oxygenase 1 (HO-1) and NAD(P)H quinone oxidoreductase 1 (NQO1), is well recognized (Nguyen et al., 2009). Recent works demonstrated that the administration of Nrf2 inducers reduced inflammatory (Redondo et al., 2017) and neuropathic pain (Negi et al., 2011; McDonnell et al., 2017a; Wang and Wang, 2017) via activation of the Nrf2/HO–1/NQO1 signaling pathway and/or inhibition of the synthesis of pro-inflammatory mediators and mitogen-activated protein kinases (MAPK) activation (Negi et al., 2011; McDonnell et al., 2017b; Redondo et al., 2017; Wang and Wang, 2017). Based on these findings and that UFP-512 exerts its cytoprotective effects in cell cultures via activation of the Nrf2/HO–1/NQO1 pathway (Cao S. et al., 2015), we evaluated whether the inhibitory effects of this DOR agonist during chronic inflammatory or neuropathic pain were also produced via activation of this antioxidant pathway and/or reduction of the inflammatory and nociceptive responses implicated in the maintenance of chronic pain. In addition and due to we previously demonstrated that the activation of Nrf2 following SFN administration increased the antinociceptive effects of DOR and MOR agonists during inflammatory and neuropathic pain (McDonnell et al., 2017a; Redondo et al., 2017; Ferreira-Chamorro et al., 2018), the potential improvement of the antinociceptive effects of UFP-512 by its coadministration with SFN in animals with inflammatory or neuropathic pain was also evaluated.

We assessed the following effects in a mouse model of inflammatory pain induced by the subplantar injection of *complete Freund’s adjuvant* (CFA) and a neuropathic pain model caused by chronic constriction of the sciatic nerve (CCI): (1) the antiallodynic and antihyperalgesic effects of the intraperitoneal administration of UFP-512 in chronic inflammatory and neuropathic pain; (2) the reversal of the antinociceptive effects of UFP-512 by the specific DOR antagonist, naltrindole, and the unspecific opioid antagonist, naloxone; (3) the antinociceptive effects of the coadministration of SFN with a low dose of UFP-512 in animals with chronic inflammatory and neuropathic pain; (4) the antidepressant effects of UFP-512 on the depressive-like behaviors linked to persistent neuropathic pain; and (5) the effects of this DOR agonist on the expression of Nrf2, HO-1, NQO1, inducible nitric oxide synthase (NOS2), phosphoinositide 3-kinase (PI3K)/protein kinase B (Akt), DOR, c-Jun N-terminal kinase (JNK), and extracellular signal regulated kinase 1/2 (ERK1/2) in the spinal cord of mice with inflammatory or neuropathic pain.

## Materials and Methods

### Animals

The animals to carry out the experimental procedures were 7-week old male C57BL/6J mice, between 21 and 25 g of weight, obtained from Envigo Laboratories (Barcelona, Spain). They were accommodated in a room with 12 h/12 h light/dark conditions, under controlled temperature of 22°C and humidity of 66%. They had free access to food and water. After a minimum of 7 days from their arrival, animals were used for doing the experiments. All experiments were conducted between 9:00 a.m. and 5:00 p.m., and executed in accordance with the National Institute of Health Guide for the Care and Use of Laboratory Animals and approved by the local Committee of Animal Use and Care of the Autonomous University of Barcelona. All efforts were made to minimize the number of animals used and their suffering.

### Induction of Inflammatory Pain

Inflammatory pain was induced by the subplantar injection of 30 μl of CFA (F5881, Sigma–Aldrich, St. Louis, MO, United States) in the right hind paw under isoflurane anesthesia (3% induction, 2% maintenance) according to the method used by our group (Leánez et al., 2009). Animals were tested at 14 days after CFA injection. Contralateral hind paws were used as controls.

### Induction of Neuropathic Pain

Neuropathic pain was caused by the chronic constriction of sciatic nerve (CCI). Nerve ligation was performed under isoflurane anesthesia (3% induction, 2% maintenance). Blunt dissection was made to segregate the biceps femoris from the gluteus superficialis on the right side. The nerve was tied by three ligatures (4/0 silk) around it, leaving 1 mm spacing and taking care to preserve epineural circulation, in accordance to the method used by our group (Hervera et al., 2013). Animals were tested at 28 days after surgery. Sham-operated mice, whose surgery was the same as described above without sciatic nerve ligation, were used as controls.

### Nociceptive Behavioral Tests

*Mechanical allodynia* was evaluated by measuring the hind paw withdrawal response to the stimulation with the von Frey filament test. Mice were placed into methacrylate cylinders (20 cm high × 9 cm in diameter) on a lifted wire grid across which von Frey filaments (North Coast Medical, Inc., San Jose, CA, United States) were applied to each hind paw by using the up/down paradigm described by Chaplan et al. (1994). After 1 h of habituation, test was started with the filament of 0.4 g and the strength of the following filament was decreased or increased in accordance with the response. The filament of 3.0 g was used as a cutoff. The threshold of response was calculated from the sequence of filament strength used during the up/down procedure utilizing an Excel program (Microsoft Iberia SRL, Barcelona, Spain), which includes curve fitting of the data. A clear paw withdrawal, licking, or shaking the paw was considered to be a nociceptive-like response.

*Thermal hyperalgesia* was evaluated by measuring hind paw withdrawal latency in response to radiant heat using the plantar test (Ugo Basile, Italy) as previously described by Hargreaves et al. (1988). Animals were placed in methacrylate cylinders (20 cm high × 9 cm diameter) on a glass surface and after 1 h of habituation, the heat source was positioned under the plantar surface of the hind paw and activated with a light beam intensity. A cut-off or 12 s was used to avoid tissue damage. The mean paw withdrawal latencies of each hind paw were determined by the average of three separated trials, taken at 5 min intervals.

In CCI-injured mice, *thermal allodynia* to a cold stimulus was also measured using the cold plate test (Ugo Basile, Italy) and according to the method described by Bennett and Xie (1988). Each animal was placed on the cold plate at 4 ± 0.5°C and the number of elevations of each hind paw was recorded for 5 min.

### Depression-Like Behavior Test

Evaluation of depressive-like behaviors was performed using the tail suspension test (TST), in which the total duration of immobility of the animal was quantified according to the method described by Steru et al. (1985), with some modifications. Briefly, mice were suspended by the tail from a horizontal wooden bar (35 cm above the floor) using adhesive tape (1 cm from the tip of the tail). The immobility time in seconds was recorded over a total period of 6 min. All the behavioral experiments were executed by an experimenter blinded to the treatment applied.

### Western Blot Analysis

Mice were euthanized by cervical dislocation at 14 days after CFA injection (inflammatory pain) or 28 days after sciatic nerve ligation (neuropathic pain). Tissues from the ipsilateral side of the lumbar section of the spinal cord were extracted, frozen in liquid nitrogen, and stored at -80°C until use. Samples of each tissue from three animals were pooled into one experimental sample to obtain the necessary protein levels to perform western blot. The protein levels of Nrf2, HO-1, NQO1, NOS2, PI3K, p-Akt, DOR, p-JNK, and p-ERK1/2 were analyzed. Tissue homogenization was made in ice cold lysis buffer (50 mM Tris Base, 150 nM NaCl, 1% NP-40, 2 mM EDTA, 1 mM phenylmethylsulfonylfluoride, 0.5 Triton X-100, 0.1% sodium dodecyl sulfate, 1 mM Na_3_VO_4_, 25 mM NaF, 0.5% protease inhibitor cocktail, and 1% phosphatase inhibitor cocktail). All reagents were obtained from Sigma–Aldrich, except for NP-40, which was acquired from Calbiochem (Darmstadt, Germany). After crude homogenate solubilization (1 h at 4°C), sonification (10 s), and centrifugation (15 min at 4°C) at 700 g, the supernatant (60 μg of total protein) was mixed with 4× laemmli loading buffer and loaded onto 4% stacking/10% separating sodium dodecyl sulfate polyacrylamide gels. Afterward, the proteins were electrophoretically transferred onto PVDF membranes (120 min). Then, they were blocked (1 h and 15 min) with phosphate-buffered saline plus 5% nonfat dry milk or Tris-buffered saline with Tween 20 plus 5% nonfat dry milk or 5% of bovine serum albumin. After that, an overnight incubation at 4°C was made with specific rabbit primary antibody anti Nrf2 (1:160; ab62352, Abcam, Cambridge, United Kingdom), NOS2 (1:160; ab204017, Abcam, Cambridge, United Kingdom), HO-1 (1:150; ab137749, Abcam, Cambridge, United Kingdom), PI3K (1:200; ab232997, Abcam, Cambridge, United Kingdom) and DOR (1:300; AB1560 Merck, Billerica, MA, United States), anti-NQO1 (1:250, N5288, Sigma–Aldrich, St. Louis, MO, United States), p-Akt (1:200; 9271, Cell Signaling Technology, Danvers, MA, United States), Akt (1:200; 9272, Cell Signaling Technology, Danvers, MA, United States), p-JNK (1:250; 9251, Cell Signaling Technology, Danvers, MA, United States), JNK (1:250; 9252, Cell Signaling Technology, Danvers, MA, United States), p-ERK1/2 (1:250; 9101, Cell Signaling Technology, Danvers, MA, United States), and ERK1/2 (1:250; 9102, Cell Signaling Technology, Danvers, MA, United States). Blots were incubated for 1 h at room temperature with a horseradish peroxidase-conjugated anti-rabbit secondary antibody (GE Healthcare, Little Chalfont, United Kingdom) to detect proteins, which were then visualized by chemiluminescence reagents (ECL kit; GE Healthcare, Little Chalfont, United Kingdom) and exposition onto hyper film (GE Healthcare, Little Chalfont, United Kingdom). The intensity of the blot was quantified by using densitometry. A rabbit anti-glyceraldehyde-3-phosphate dehydrogenase (GAPDH) antibody (1:5000; ABS16, Merck, Billerica, MA, United States) was used as a loading control.

### Experimental Procedure

First of all, in both inflammatory and neuropathic pain models, behavioral baseline responses were measured in the following order: von Frey filaments, plantar test, and/or cold plate test. At 14 days after CFA injection or at 28 days after surgery, the antinociceptive effects produced by 1, 3, 10, 20, and 30 mg/kg of UFP-512 were tested according to the sequence described above. UFP-512 was injected intraperitoneally and the nociceptive responses were tested 1 h later in accordance to our previous pilot studies and another work (Aguila et al., 2007). In the inflammatory pain model, the contralateral hind paw was used as a control (*n* = 6 animals per dose), while in neuropathic pain model, sham-operated animals were used as control (*n* = 6 animals per dose).

The reversibility of the antinociceptive effects of 30 mg/kg UFP-512, which produces the maximal antiallodynic and antihyperalgesic effects after peripheral inflammation and sciatic nerve injury, with the subcutaneous administration of the specific DOR antagonist, naltrindole (4 mg/kg) and the unspecific opioid antagonist, naloxone (1 mg/kg) was evaluated 14 days after CFA injection or 28 days after surgery (*n* = 6 animals per group). The doses of all tested antagonists were selected according to previous works (Hervera et al., 2013; Carcolé et al., 2014).

In order to study the possible enhancement of the analgesic effects of UFP-512 induced by SFN, in other set of experiments we evaluated the antiallodynic and antihyperalgesic effects produced by the intraperitoneal administration of a low dose of UFP-512 (1 mg/kg) combined with 10 mg/kg of SFN at day 14 after CFA injection or 28 days after surgery. The dose of SFN was selected in accordance to other studies (Redondo et al., 2017; Ferreira-Chamorro et al., 2018) and the dose of UFP-512 was chosen from the dose–response curves performed in this study, as the ones that produced minimal inhibitory effects in both pain models. SFN was intraperitoneally administrated 2 h before UFP-512 injection and the nociceptive responses were evaluated 1 h later, in accordance to our previous works (Redondo et al., 2017; Ferreira-Chamorro et al., 2018) (*n* = 6 animals per group).

In animals with neuropathic pain, at day 28 after CCI, the antidepressant effects produced by 1 mg/kg of UFP-512 in the TST were also assessed. Sham-operated mice were used as control (*n* = 8 animals per group).

Finally, the effects of UFP-512 on the expression of Nrf2, HO-1, NQO1, NOS2, PI3K, p-Akt, DOR, p-JNK, and p-ERK1/2 in the ipsilateral site of spinal cords of animals with chronic peripheral inflammation or sciatic nerve injury were evaluated by using western blot. Naive and sham-operated mice treated with vehicle were used as controls for CFA-injected animals and CCI-injured mice, respectively (*n* = 4 samples per group).

### Drugs

UFP-512 was synthetized by Balboni et al. (2002), SFN was acquired from Merck Chemicals and Life Science S.A.U (Madrid, Spain), and naltrindole and naloxone were purchased in Sigma–Aldrich (St. Louis, MO, United States). UFP-512 was dissolved in saline solution (0.9%) and intraperitoneally administered at 1, 3, 10, 20, and 30 mg/kg. SFN was dissolved in dimethylsulfoxide (1% in saline solution 0.9%) and intraperitoneally administered at 10 mg/kg. Naltrindole and naloxone were also diluted in saline solution (0.9%) and subcutaneously administered at 4 and 1 mg/kg, respectively. All drugs were prepared daily just before use and administered in a final volume of 10 ml/kg, at 1 h (UFP-512), 3 h (SFN), and 30 min (naltrindole and naloxone) before testing in accordance to our previous pilot studies and other works (Aguila et al., 2007; Hervera et al., 2013; Carcolé et al., 2014; Redondo et al., 2017; Ferreira-Chamorro et al., 2018). For each group treated with a drug, the respective control group received the same volume of corresponding vehicle.

### Statistical Analysis

Data are expressed as mean ± standard error of the mean (SEM). All the statistical analysis was carried out using the SPSS program (version 17 for windows, IBM España, Madrid, Spain).

In both pain models and for each nociceptive behavioral test, the comparison of the effects produced by the intraperitoneal administration of different doses of UFP-512 or saline was evaluated by using a one-way ANOVA, followed by the Student–Newman–Keuls test.

In all experiments, the antiallodynic effects in the von Frey filaments and the antihyperalgesic effects in the plantar test were expressed as the percentage of maximal possible effect, where the test latencies predrug (baseline) and postdrug administration are compared and calculated according to the following equation:

Maximal possible effect (%) = [(drug–baseline)/(cut-off–baseline)] × 100.

In the cold plate test, the inhibitory effects were calculated according to the following equation:

Inhibition (%) = [(paw elevations number at baseline–paw elevations number after drug)/paw elevations number at baseline] × 100.

For each test, the reversal of the systemic antinociceptive effects produced by UFP-512 with naltrindole or naloxone as well as the effects of these antagonists administered alone were analyzed by using a one-way ANOVA, followed by the Student–Newman–Keuls test.

In both pain models, the comparison of the antiallodynic and antihyperalgesic effects produced by the coadministration of SFN with UFP-512 was assessed by using a one-way ANOVA, followed by the Student–Newman–Keuls test.

The analysis of the antidepressant actions of UFP-512 in animals with neuropathic pain was performed by using a two-way ANOVA, followed by the corresponding one-way ANOVA, and the Student–Newman–Keuls test.

The effects of UFP-512 on the protein levels of Nrf2, HO-1, NQO1, NOS2, PI3K, p-Akt, DOR, p-JNK, and p-ERK1/2 in the spinal cord of mice with inflammatory or neuropathic pain were analyzed by using a one-way ANOVA, followed by Student–Newman–Keuls test. A *P <* 0.05 was considered significant.

## Results

### Effects of UFP-512 on Mechanical Allodynia and Thermal Hyperalgesia Induced by Inflammatory Pain

**FIGURE 1:**
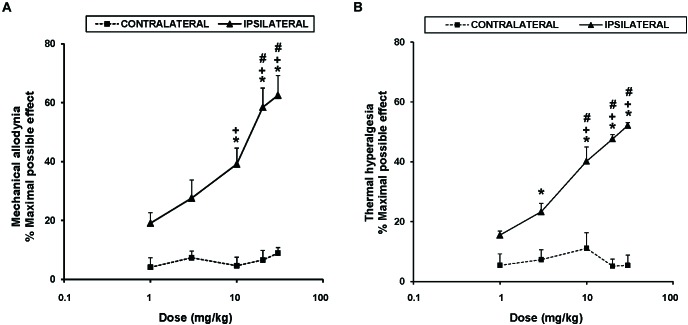
Effects of UFP-512 on the mechanical allodynia and thermal hyperalgesia induced by CFA. The effects of acute intraperitoneal administration of different doses (logarithmic axis) of UFP-512 on the mechanical allodynia **(A)** and thermal hyperalgesia **(B)** induced by CFA in ipsilateral (continuous lines) and contralateral hind paws (discontinuous lines) are shown. UFP-512 was administered 1 h before testing. In both panels and for each dose assessed, ^∗^ indicates significant differences vs. their respective effects in the contralateral paw, ^+^ indicates significant differences vs. the effect produced by 1 mg/kg of UFP-512 in the ipsilateral paw and ^#^ indicates significant differences vs. the effect produced by 3 mg/kg of UFP-512 in the ipsilateral paw (*P <* 0.05; one-way ANOVA, followed by the Student–Newman–Keuls test). In both tests, data are expressed as mean values of maximal possible effect (%) ± SEM; *n* = 6 animals per dose.

To study the antinociceptive actions of UPF-512 during inflammatory pain, we evaluated the effects of acute intraperitoneal administration of 1, 3, 10, 20, and 30 mg/kg UPF-512 on CFA-induced mechanical allodynia and thermal hyperalgesia 14 days after injection. Our results showed that UFP-512 administration 1 h before testing inhibited CFA-induced mechanical allodynia (Figure 1A) and thermal hyperalgesia (Figure 1B) in a dose-dependent manner and reached a maximum effect at 30 mg/kg.

The mechanical antiallodynic effects produced by 10, 20, and 30 mg/kg UFP-512 in the ipsilateral paw of CFA-injected mice were significantly greater than the effects produced by each respective dose in the contralateral paw (P < 0.001, one-way ANOVA, followed by the Student–Newman–Keuls test). Moreover, the antiallodynic effects produced by the administration of high doses (10, 20, or 30 mg/kg) of UFP-512 were significantly higher than the effects produced by lower doses (1 and/or 3 mg/kg) of this DOR agonist in the ipsilateral paw (P < 0.001, one-way ANOVA, followed by the Student–Newman–Keuls test).

The thermal antihyperalgesic effects produced by 3, 10, 20, and 30 mg/kg UFP-512 in the ipsilateral paw of CFA-injected mice were also greater than the effects produced by each respective dose in the contralateral paw (*P <* 0.001, one-way ANOVA, followed by the Student–Newman–Keuls test). The antihyperalgesic actions induced by 10, 20, or 30 mg/kg UFP-512 in the ipsilateral paw were also higher than the effects produced by 1 and 3 mg/kg of UFP-512 in the same paw.

The intraperitoneal administration of saline did not elicit any antinociceptive effects in the contralateral or ipsilateral paw of CFA-injected mice in either test (data not shown).

### Effects of UFP-512 on the Mechanical Allodynia, Thermal Hyperalgesia, and Thermal Allodynia Induced by Sciatic Nerve Injury

**FIGURE 2:**
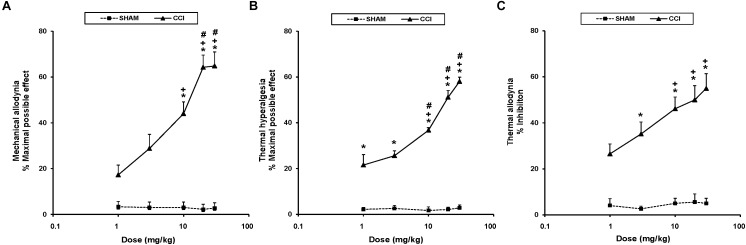
Effects of UFP-512 on the mechanical allodynia, thermal hyperalgesia, and thermal allodynia induced by CCI. The effects of the intraperitoneal acute administration of different doses (logarithmic axis) of UFP-512 on the mechanical allodynia **(A)**, thermal hyperalgesia **(B)**, and thermal allodynia **(C)** induced by sciatic nerve injury in the ipsilateral hind paw of CCI-injured (continuous lines) or sham-operated mice (discontinuous lines) are represented. UFP-512 was administered 1 h before testing. For each test and dose evaluated, ^∗^ indicates significant differences vs. their respective effects in sham-operated mice, ^+^ indicates significant differences vs. the effects produced by 1 mg/kg of UFP-512 in CCI-injured mice, and ^#^ indicates significant differences vs. the effects produced by 3 mg/kg of UFP-512 in CCI-injured mice (*P <* 0.05; one-way ANOVA, followed by the Student–Newman–Keuls test). Data are expressed as mean values of maximal possible effect (%) for mechanical allodynia and thermal hyperalgesia, and inhibition (%) for thermal allodynia ± SEM; *n* = 6 animals per dose.

To study the antinociceptive actions of UPF-512 during neuropathic pain, we evaluated the effects of the acute intraperitoneal administration of UFP-512 at 1, 3, 10, 20, and 30 mg/kg on the mechanical allodynia, thermal hyperalgesia, and thermal allodynia induced by sciatic nerve injury 28 days after surgery. Our results indicated that UFP-512 administration 1 h before testing inhibited CCI-induced mechanical allodynia (Figure 2A), thermal hyperalgesia (Figure 2B), and thermal allodynia (Figure 2C) in a dose-dependent manner, and 30 mg/kg produced the maximum effect.

Therefore, the mechanical antiallodynic effects produced by 10, 20, and 30 mg/kg of UFP-512 in CCI-injured mice were greater than the effects produced by each respective dose in sham-operated mice and the effects produced by 1 and 3 mg/kg of this DOR agonist in CCI-injured mice (*P <* 0.001; one-way ANOVA, followed by the Student–Newman–Keuls test).

The thermal antihyperalgesic effects produced by 1, 3, 10, 20, and 30 mg/kg of UFP-512 in CCI-injured animals were higher than the effects produced by each respective dose in sham-operated mice (*P <* 0.001; one-way ANOVA, followed by the Student–Newman–Keuls test). The antihyperalgesic effects of 10, 20, and 30 mg/kg of UFP-512 were also higher than the effects of 1 and 3 mg/kg in CCI-injured mice (*P <* 0.001; one-way ANOVA, followed by the Student–Newman–Keuls test).

Similar effects were observed for thermal allodynia. The antiallodynic actions produced by 3, 10, 20, and 30 mg/kg of UFP-512 in CCI-injured mice were greater than the effects produced by these doses in sham-operated mice (*P <* 0.001; one-way ANOVA, followed by the Student–Newman–Keuls test). The antiallodynic effects produced by high doses (10, 20, and 30 mg/kg) of UFP-512 in CCI-injured mice were greater than the effects produced by 1 mg/kg of this drug in these animals.

Treatment with UFP-512 did not affect the contralateral paw of CCI-injured or sham-operated mice in any experiment (data not shown). The intraperitoneal administration of saline did not elicit any antinociceptive effect in the contralateral or ipsilateral paw of CCI-injured or sham-operated mice (data not shown).

### Reversal of the Antinociceptive Effects of UFP-512 in Animals With Inflammatory or Neuropathic Pain

**FIGURE 3:**
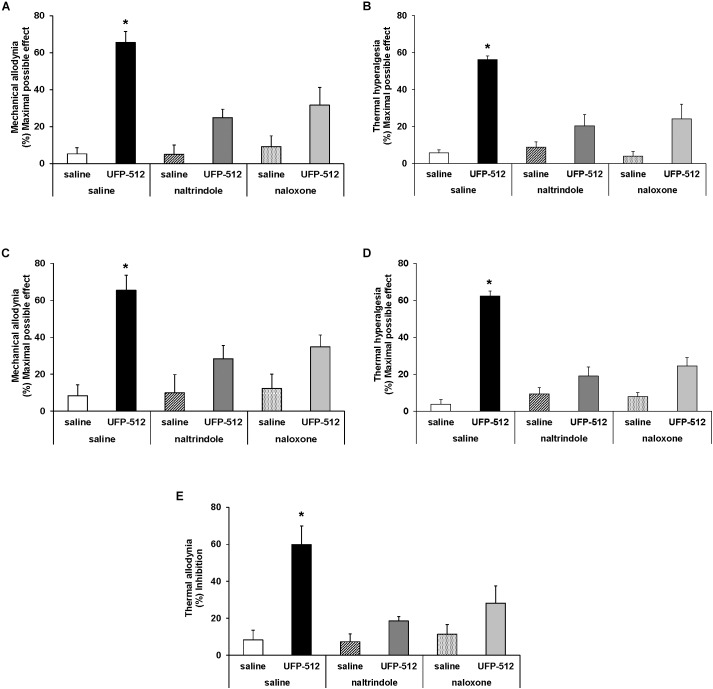
Reversion of the antiallodynic and antihyperalgesic effects of UFP-512. Reversal of the effects induced by 30 mg/kg UFP-512 on the mechanical allodynia **(A)** and thermal hyperalgesia **(B)** induced by CFA, and on the mechanical allodynia **(C)**, thermal hyperalgesia **(D)**, and thermal allodynia **(E)** caused by CCI, in ipsilateral paws, with the administration of the specific DOR antagonist, naltrindole (4 mg/kg), and the unspecific opioid antagonist, naloxone (1 mg/kg) are shown. The effects of vehicle, naltrindole (4 mg/kg), or naloxone (1 mg/kg) administered alone are also represented. UFP-512 was intraperitoneally injected 1 h before testing while both naltrindole and naloxone were subcutaneously injected 30 min before testing. For each test, ^∗^ represents significant differences compared to the other groups (*P <* 0.05; one-way ANOVA, followed by the Student–Newman–Keuls test). Data are expressed as mean values of maximal possible effect (%) for mechanical allodynia and thermal hyperalgesia, and inhibition (%) for thermal allodynia ± SEM (six animals for each group).

To evaluate the specificity of the antinociceptive effects produced by a high dose of UFP-512 (30 mg/kg) during inflammatory and neuropathic pain, mice were administered with the specific DOR antagonist, naltrindole (4 mg/kg) and the unspecific opioid antagonist, naloxone (1 mg/kg). UPF was intraperitoneally administered 1 h before testing, and naltrindole and naloxone were subcutaneously administered 30 min before testing. Our results showed that the mechanical antiallodynic (Figure 3A) and thermal antihyperalgesic (Figure 3B) effects produced by UFP-512 in the ipsilateral paw of CFA-injected animals and the mechanical antiallodynic (Figure 3C), thermal antihyperalgesic (Figure 3D), and thermal antiallodynic (Figure 3E) effects produced by this drug in the ipsilateral paw of sciatic nerve-injured mice were reversed by the subcutaneous administration of naltrindole and naloxone (*P <* 0.005; one-way ANOVA, followed by the Student–Newman–Keuls test).

The subcutaneous administration of both antagonists administered alone in CFA-injected animals (Figure 3A,B) and sciatic nerve-injured mice (Figure 3C–E) did not produce any significant effect in the ipsilateral paw of these animals. Naltrindole and naloxone administration alone also did not produce any effect in the contralateral paw of CFA-injected mice or sciatic nerve-injured mice or in the ipsilateral and contralateral paw of sham-operated mice (data not shown).

### Effect of UFP-512 on the Expression of Nrf2, HO-1, NQO1, and NOS2 in the Spinal Cords of Animals With Inflammatory Pain

To evaluate the possible mechanisms of the antinociceptive effects of UFP-512 during inflammatory pain, the expression of Nrf2, HO-1, NQO1, and NOS2 in the spinal cords of CFA-injected mice treated with 30 mg/kg UFP-512 or vehicle was assessed 14 days after CFA injection. We used naïve mice treated with vehicle as controls in these experiments.

**FIGURE 4:**
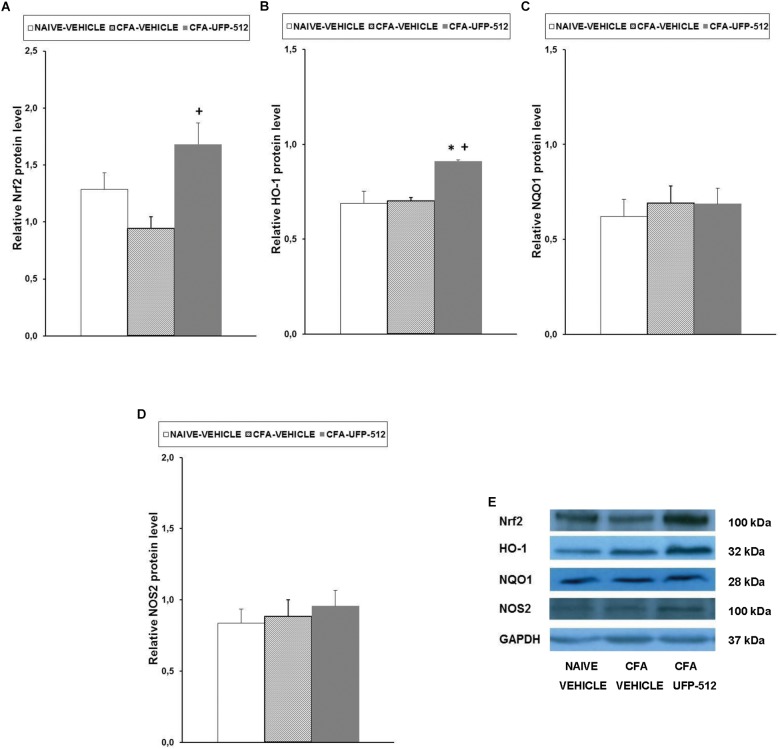
Effects of UFP-512 on the expression of Nrf2, HO-1, NQO1, and NOS2 in the spinal cords of animals with peripheral inflammation. Protein levels of Nrf2 **(A)**, HO-1 **(B)**, NQO1 **(C)**, and NOS2 **(D)** in the ipsilateral site of the spinal cords of CFA-injected mice treated with UFP-512 (CFA-UFP-512) or vehicle (CFA-vehicle) are represented. Controls corresponding to naive mice treated with vehicle (naive-vehicle) are also shown. For each protein, ^∗^ indicates significant differences vs. naïve mice treated with vehicle and + indicates significant differences vs. CFA-injected mice treated with vehicle (*P <* 0.05, one-way ANOVA, followed by the Student–Newman–Keuls test). Representative examples of western blots for Nrf2 (100 kDa), HO-1 (32 kDa), NQO1 (28 kDa), and NOS2 (100 kDa) proteins in which GAPDH (37 kDa) was used as a loading control are also shown **(E)**. Results are expressed as mean ± SEM; *n* = 4 samples per group.

Our results showed that peripheral inflammation did not alter the spinal cord expression of Nrf2, but Nrf2 protein levels increased significantly in CFA-injected mice treated with UFP-512 (*P <* 0.022, one-way ANOVA vs. CFA-injected mice treated with vehicle) (Figure 4A). Peripheral inflammation did not alter the expression of HO-1 in the spinal cord compared to naïve animals treated with vehicle, but HO-1 levels increased significantly in CFA-injected mice treated with UFP-512 (*P <* 0.011, one-way ANOVA vs. naive and CFA-injected mice treated with vehicle) (Figure 4B). No significant differences in NQO1 (Figure 4C) or NOS2 (Figure 4D) were observed between the three evaluated groups.

### Effect of UFP-512 on the Expression of Nrf2, HO-1, NQO1, and NOS2 in the Spinal Cords of Animals With Neuropathic Pain

To assess the possible mechanisms of the antinociceptive effects of UFP-512 during neuropathic pain, the expression of Nrf2, HO-1, NQO1, and NOS2 in the spinal cords of CCI-injured mice treated with 30 mg/kg of this DOR agonist or vehicle was assessed 28 days after surgery.

**FIGURE 5:**
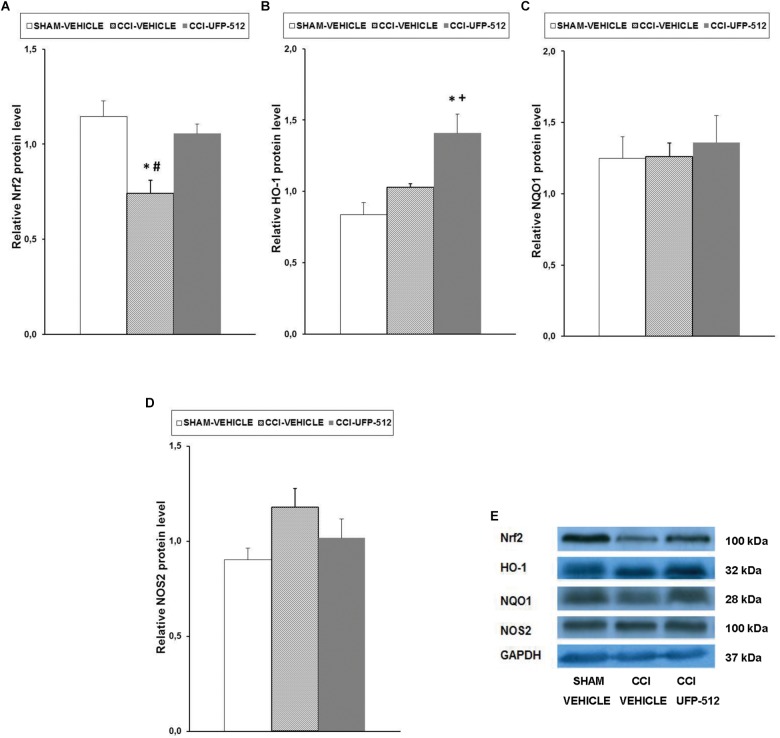
Effects of UFP-512 on the expression of Nrf2, HO-1, NQO1, and NOS2 in spinal cords of animals with neuropathic pain. Protein levels of Nrf2 **(A)**, HO-1 **(B)**, NQO1 **(C)**, and NOS2 **(D)** in the ipsilateral site of spinal cords of CCI-injured mice treated with UFP-512 (CCI-UFP-512) or vehicle (CCI-vehicle) are represented. Controls corresponding to sham-operated mice treated with vehicle (sham-vehicle) are also shown. For each protein, ^∗^ indicates significant differences vs. sham-operated mice treated with vehicle, ^+^ indicates significant differences vs. CCI-injured mice treated with vehicle, and ^#^ indicates significant differences vs. CCI-injured mice treated with UFP-512 (*P <* 0.05, one-way ANOVA, followed by the Student–Newman–Keuls test). Representative examples of western blots for Nrf2 (100 kDa), HO-1 (32 kDa), NQO1 (28 kDa), and NOS2 (100 kDa) proteins in which GAPDH (37 kDa) was used as a loading control are also shown **(E)**. Results are expressed as mean ± SEM; *n* = 4 samples per group.

Reduction in Nrf2 protein levels in the spinal cord following CCI (*P <* 0.007, one-way ANOVA, compared to sham-operated mice treated with vehicle) was completely normalized with UFP-512 administration (Figure 5A). CCI did not alter the protein levels of HO-1, but treatment with UFP-512 significantly increased HO-1 expression in CCI-injured mice (*P <* 0.008, one-way ANOVA compared to CCI-injured and sham-operated mice treated with vehicle) (Figure 5B). Neither CCI nor UFP-512 treatment altered NQO1 (Figure 5C) or NOS2 (Figure 5D) expression compared to sham-operated-mice.

### Effect of UFP-512 on the Expression of PI3K, p-Akt, DOR, p-JNK, and p-ERK1/2 in the Spinal Cords of Animals With Inflammatory Pain

**FIGURE 6:**
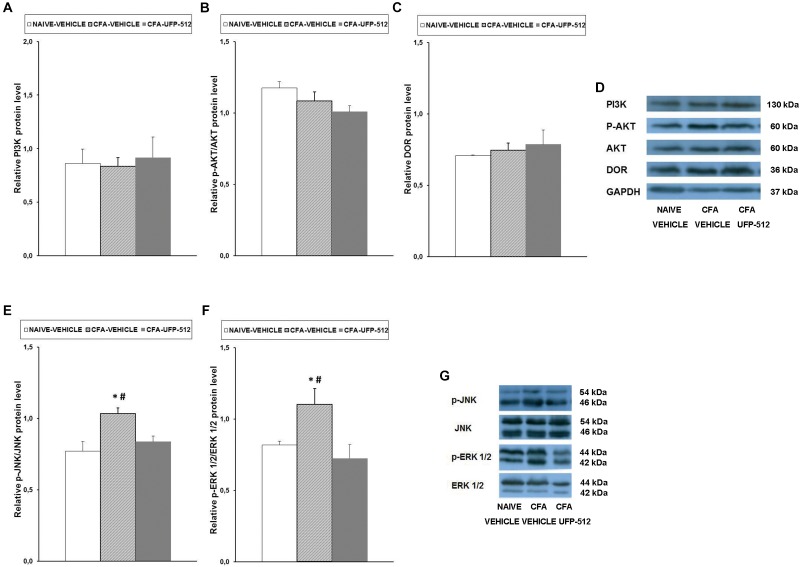
Effects of UFP-512 on the expression of PI3K, p-Akt, DOR, p-JNK, and p-ERK 1/2 in the spinal cords of animals with peripheral inflammation. Protein levels of PI3K **(A)**, p-Akt **(B)**, DOR **(C)**, p-JNK **(E)**, and p-ERK 1/2 **(F)** in the ipsilateral site of spinal cords of CFA-injected mice treated with UFP-512 (CFA-UFP-512) or vehicle (CFA-vehicle) are represented. Controls corresponding to naive mice treated with vehicle (naive-vehicle) are also shown. For each protein, ^∗^ indicates significant differences vs. naive-vehicle treated mice and ^#^ indicates significant differences vs. CFA-injected mice treated with UFP-512 (*P <* 0.05, one-way ANOVA, followed by the Student–Newman–Keuls test). Representative examples of western blots for PI3K (130 kDa), p-Akt (60 kDa), Akt (60 kDa), DOR (36 kDa), and GAPDH (37 kDa) are shown in **(D)** and for p-JNK/total JNK protein (46–54 kDa) and p-ERK 1/2/total ERK 1/2 (42–44 kDa) in **(G)**. Phosphorylated proteins are expressed relative to their corresponding total proteins while the rest are relative to GAPDH. Results are expressed as mean ± SEM; *n* = 4 samples per group.

To evaluate the possible effects of UFP-512 treatment on PI3K/p-AKT, MAPK (JNK and ERK1/2), and DOR expression during inflammatory pain, the effects of this DOR agonist on the protein levels of PI3K (Figure 6A), p-Akt (Figure 6B), DOR (Figure 6C), p-JNK (Figure 6E), and p-ERK1/2 (Figure 6F) in the spinal cords of CFA-injected mice treated with UFP-512 were also assessed.

Our results did not show any significant alterations of PI3K, p-AKT, or DOR expression following inflammation or treatment. In contrast, the significant increase in spinal cord protein levels of p-JNK (*P <* 0.027, one-way ANOVA vs. naïve mice treated with vehicle) and p-ERK1/2 (*P <* 0.024, one-way ANOVA vs. naïve mice treated with vehicle) induced by peripheral inflammation were reversed with UFP-512 treatment.

### Effect of UFP-512 on the Expression of PI3K, p-Akt, DOR, p-JNK, and p-ERK1/2 in the Spinal Cords of Animals With Neuropathic Pain

**FIGURE 7:**
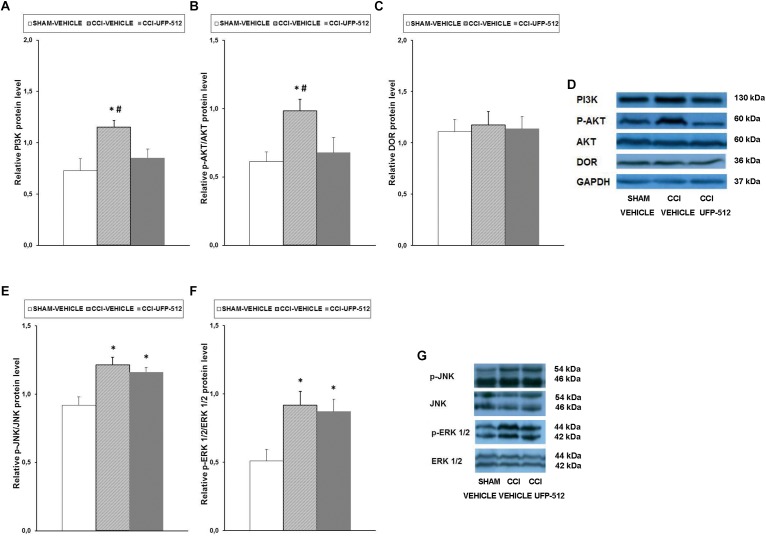
Effects of UFP-512 on the expression of PI3K, p-Akt, DOR, p-JNK, and p-ERK 1/2 in the spinal cords of animals with neuropathic pain. Protein levels of PI3K **(A)**, p-Akt **(B)**, DOR **(C)**, p-JNK **(E)**, and p-ERK 1/2 **(F)** in the ipsilateral site of the spinal cord from CCI-injured mice treated with UFP-512 (CCI-UFP-512) or vehicle (CCI-vehicle) are represented. Controls corresponding to sham-operated mice treated with vehicle (sham-vehicle) are also shown. For each protein,^∗^ indicates significant differences vs. sham-operated mice treated with vehicle and ^#^ indicates significant differences vs. CCI-injured mice treated with UFP-512 (*P <* 0.05, one-way ANOVA, followed by the Student–Newman–Keuls test). Representative examples of western blots for PI3K (130 kDa), p-Akt (60 kDa), Akt (60 kDa), DOR (36 kDa), and GAPDH (37 kDa) are shown in **(D)** and for p-JNK/total JNK protein (46–54 kDa) and p-ERK 1/2/total ERK 1/2 (42–44 kDa) in **(G)**. Phosphorylated proteins are expressed relative to their corresponding total proteins while the rest are relative to GAPDH. Results are expressed as mean ± SEM; *n* = 4 samples per group.

To assess the possible effects of UFP-512 treatment on PI3K/p-AKT, MAPK (JNK and ERK1/2), and DOR expression during neuropathic pain, the effects of this DOR agonist on the protein levels of PI3K (Figure 7A), p-Akt (Figure 7B), DOR (Figure 7C), p-JNK (Figure 7E), and p-ERK1/2 (Figure 7F) in the spinal cords of CCI-injured mice treated with UFP-512 were also investigated.

Our data showed that, in contrast to the effects of peripheral inflammation, sciatic nerve injury increased the spinal protein levels of PI3K (*P* < 0.027, one-way ANOVA compared to sham-operated mice treated with vehicle), and UFP-512 inhibited this increase. The nerve injury-induced increased expression of p-Akt (*P* < 0.029, one-way ANOVA compared to sham-operated mice treated with vehicle) was also reversed by UFP-512 administration. No changes in the protein levels of DOR were observed in the three groups tested.

Our results also demonstrated that, similar to inflammatory pain, sciatic nerve injury augmented the expression of p-JNK (*P <* 0.007, one-way ANOVA vs. sham-operated mice treated with vehicle) and p-ERK1/2 (*P <* 0.026; one-way ANOVA vs. sham-operated mice treated with vehicle) in the spinal cord. However, in this case, treatment with UFP-512 did not inhibit this increased expression.

### Effects of the Coadministration of SFN on the Antinociceptive Actions of UFP-512 in Animals With Inflammatory or Neuropathic Pain

To study the antinociceptive actions produced by the administration of SFN plus UFP-512 during chronic pain, the effects of the intraperitoneal administration of 10 mg/kg of SFN combined with a low dose (1 mg/kg) of UFP-512 against the mechanical allodynia and thermal hyperalgesia caused by peripheral inflammation or sciatic nerve injury were evaluated.

**FIGURE 8:**
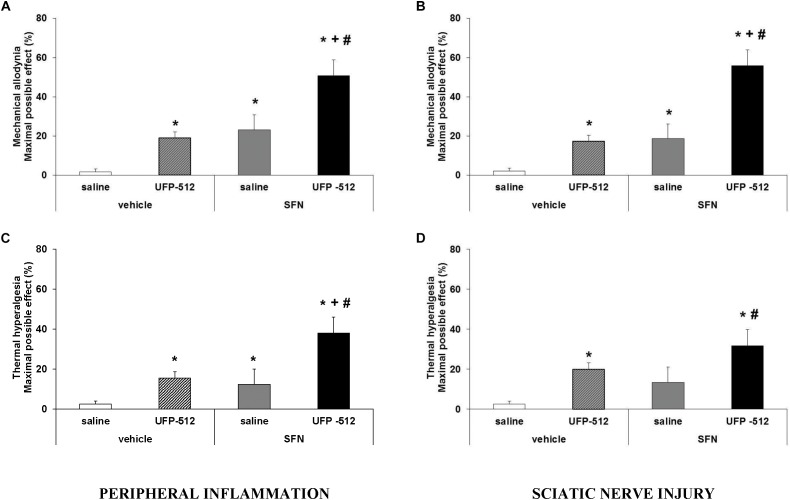
Effects of the coadministration of UFP-512 with SFN on the mechanical allodynia and thermal hyperalgesia induced by peripheral inflammation or sciatic nerve injury. Mechanical antiallodynic and thermal antihyperalgesic effects produced by 10 mg/kg SFN and 1 mg/kg UFP-512 intraperitoneally administered alone or combined in animals with inflammatory **(A, C)** or neuropathic pain **(B, D)** are shown. SFN and UFP-512 were administered at 3 and 1 h before testing, respectively. For each test, ^∗^ indicates significant differences vs. its respective animals treated with vehicle plus saline, ^+^ indicates significant differences vs. its respective animals treated with vehicle plus UFP-512, and ^#^ indicates significant differences vs. its respective animals treated with SFN plus saline (*P <* 0.05, one-way ANOVA, followed by the Student–Newman–Keuls test). Data are expressed as mean values of maximal possible effect (%) for mechanical allodynia and thermal hyperalgesia ± SEM; *n* = 6 animals per group.

For mechanical allodynia, the coadministration of SFN with UFP-512 produced an antiallodynic effect that was significantly higher than either UFP-512 or SFN administration alone in animals with inflammatory (Figure 8A; *P <* 0.001, one-way ANOVA, followed by the Student–Newman–Keuls test) or neuropathic pain (Figure 8B; P < 0.001, one-way ANOVA, followed by the Student–Newman–Keuls test).

Regarding thermal hyperalgesia, our data showed that the administration of SFN plus UFP-512 produced an antihyperalgesic effect in CFA-injected mice that was higher than these drugs administered alone (*P <* 0.001, one-way ANOVA, followed by the Student–Newman–Keuls test) (Figure 8C). The combination of both drugs tended to increase the antihyperalgesic effects produced by each drug administered separately in CCI mice (Figure 8D), but the combination effects only differed significantly when compared to the effects produced by SFN or vehicle administered alone (*P <* 0.001, one-way ANOVA, followed by the Student–Newman–Keuls test).

### The Effects of UFP-512 on the Depressive-Like Behaviors Associated With Neuropathic Pain

To assess the possible antidepressant effects of UFP-512 in animals with depressive-like behaviors associated with persistent neuropathic pain, the effects of the intraperitoneal administration of 1 mg/kg UFP-512 in CCI-injured mice were evaluated 28 days after surgery using the TST 1 h after injection.

**FIGURE 9:**
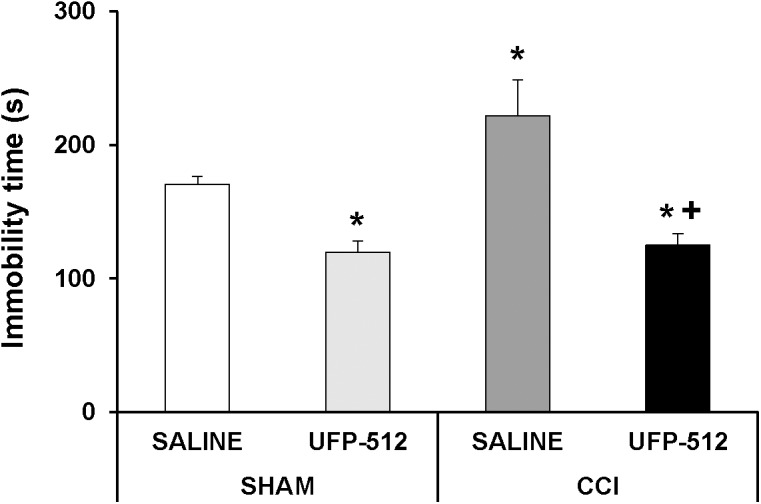
Effects of UFP-512 on the depressive-like behaviors associated with neuropathic pain. Immobility time in seconds (s) for CCI-injured and sham-operated mice treated with 1 mg/kg of UFP-512 or saline evaluated at 28 days after surgery in TST are represented. UFP-512 was administered 1 h before testing. In all groups, ^∗^ indicates significant differences vs. sham-operated mice treated with saline and ^+^ indicates significant differences vs. CCI-injured mice treated with saline (*P <* 0.05, one-way ANOVA, followed by the Student–Newman–Keuls test). Results are expressed as mean ± SEM; *n* = 8 animals for each experimental group.

Two-way ANOVA revealed a significant effect of the surgery (*P <* 0.049) and treatment (*P <* 0.001). The increased immobility time in CCI-injured animals (Figure 9; *P <* 0.001, one-way ANOVA vs. sham-operated mice treated with saline) was significantly reduced with UFP-512 administration. This treatment also reduced the immobility time in sham-operated mice (*P <* 0.001, one-way ANOVA vs. sham-operated mice treated with saline). Therefore, our results confirmed the antidepressant effects of this DOR agonist in animals without pain and further demonstrated its antidepressant activity in mice with depressive-like behavior associated with persistent neuropathic pain.

## Discussion

The present study revealed that the administration of UFP-512 diminished the allodynia and hyperalgesia caused by chronic peripheral inflammation and increased Nrf2-HO-1 protein levels in the spinal cords of these animals. This DOR agonist also inhibited the mechanical and thermal allodynia and thermal hyperalgesia induced by sciatic nerve ligation and normalized and/or increased the spinal cord expression of Nrf2 and HO-1. The antinociceptive effects of UFP-512 were reversed by the administration of the specific DOR antagonist, naltrindole, and the unspecific opioid antagonist, naloxone. UFP-512 blocked the phosphorylation of JNK and ERK1/2 induced by peripheral inflammation in CFA-injected mice, and this drug inhibited activation of PI3K/Akt in CCI animals, but it did not alter CCI-induced JNK and ERK1/2 phosphorylation. UFP-512 inhibited the depressive-like behavior associated with persistent neuropathic pain, and its coadministration with SFN improved its antiallodynic effects in animals with inflammatory or neuropathic pain.

Our results revealed, for the first time, that treatment with UFP-512 inhibited the allodynia and hyperalgesia caused by peripheral inflammation or sciatic nerve injury in a dose-dependent manner. These results are consistent with the analgesic effects of other DOR agonists, such as DPDPE or SNC-80, during inflammatory (Carcolé et al., 2014) or neuropathic pain caused by nerve injury (Hervera et al., 2010) or diabetes (Castany et al., 2016; McDonnell et al., 2017a). Our data also revealed that analogous doses of UFP-512 produced comparable antiallodynic and antihyperalgesic effects in animals with inflammatory or neuropathic pain. A dose of 30 mg/kg of UFP-512, for example, similarly inhibited the mechanical allodynia (65%) and thermal hyperalgesia (60%) in both pain models. These results are consistent with the similar effectivity showed by other DOR agonists in the inhibition of acute inflammatory (3 h) and neuropathic pain (16 days after surgery) (Obara et al., 2009) and further demonstrated that this analogous efficacy was also observed during chronic inflammatory (14 days after CFA injection) and neuropathic pain (28 days after CCI). Therefore, and considering the few side effects of this DOR agonist compared to MOR agonists (Aguila et al., 2007; Fernández-Dueñas et al., 2007; Hervera et al., 2012; Bodnar, 2016), the present results support the use of UFP-512 as an interesting treatment for chronic pain management.

The present study only evaluated the antinociceptive effects of systemic UFP-512 in male mice. However, the peripheral administration of other DOR agonists, such as DPDPE, also inhibits inflammatory and neuropathic pain (Hervera et al., 2010, 2013; Carcolé et al., 2014) and 10-fold higher dose of DPDPE is required in female rats to produce analogous anti-hyperalgesic responses that achieved in male rats, revealing sex differences in the peripheral effects of this DOR agonist (Saloman et al., 2011). Therefore, the potential peripheral antinociceptive effects of UFP-512 in male and female rodents need to be investigated.

The reversion of the antiallodynic and antihyperalgesic effects of UFP-512 during inflammatory or neuropathic pain was evaluated by measuring its effects in the presence of the specific DOR antagonist, naltrindole, and the unspecific opioid antagonist, naloxone. Both antagonists reversed the antinociceptive effects of UFP-512, which suggests that these effects are mediated via DOR, although we cannot exclude a contribution of MOR to the antinociceptive effects of UFP-512 in these experimental conditions. In accordance to our results, previous studies of Balboni et al. (2002, 2008) also reported that the antinociceptive effects of centrally administered UFP-512 were DOR-mediated based on the reversal by the specific DOR antagonist, naltrindole.

Notably, previous studies in cell cultures showed that UFP-512 stimulated the Nrf2 transcription factor (Cao S. et al., 2015), which is a key mechanism of defense against oxidative stress and inflammatory processes. Our results further demonstrated that UFP-512 also enhanced the expression of Nrf2 and HO-1 in the spinal cords of mice with peripheral inflammation and normalized the decreased expression of Nrf2 induced by nerve injury or enhanced the protein levels of HO-1 in the spinal cord of sciatic nerve-injured mice. These latter results confirmed the long-lasting state of oxidative stress that is induced by sciatic nerve ligation in the spinal cord (Riego et al., 2018) and revealed, for the first time, the ability of UFP-512 to normalize this effect *in vivo.* Moreover and tacking account that several Nrf2 inducers produce antinociceptive effects during chronic pain (Negi et al., 2011; McDonnell et al., 2017a; Redondo et al., 2017), the augment or normalization of Nrf2 expression in the spinal cord following systemic UFP-512 administration supports the hypothesis that Nrf2 might be involved in the antinociceptive effects of UFP-512 in the presence of chronic inflammatory or neuropathic pain. The substantial improvement in the antiallodynic actions of UFP-512 induced by SFN in animals with inflammatory or neuropathic pain suggests that the coadministration of Nrf2 activators with UFP-512 is another interesting approach for the treatment of chronic pain because the maximal antinociceptive effect (approx. 60%) was induced using a small dose of UFP-512, minimizing side effects. In accordance to our findings, other studies also demonstrated that SFN potentiated the analgesic effects of specific DOR agonists, such as DPDPE and SNC-80, in animals with diabetic neuropathy (McDonnell et al., 2017a) and improved the local antiallodynic and/or antihyperalgesic effects of MOR agonists, for example morphine, under inflammatory and neuropathic pain conditions (Redondo et al., 2017; Ferreira-Chamorro et al., 2018).

The expression of HO-1, which is a relevant antioxidant enzyme, was also increased by UFP-512 in the spinal cord of mice with inflammatory or neuropathic pain. This result suggests that this isoenzyme plays a role in the antinociceptive effects produced by this DOR agonist. Others studies also demonstrated that the administration of HO-1 inducer compounds, such as cobalt protoporphyrin IX, improved the antinociceptive activity of other DOR agonists during inflammatory pain (Carcolé et al., 2014) or diabetic neuropathy (Castany et al., 2016). In summary, these findings suggest that the Nrf2/HO-1 pathway is involved in the analgesic actions of UFP-512 during chronic pain.

Curiously, and in contrast to the *in vitro* effects (Cao S. et al., 2015), UFP-512 treatment did not alter the unchanged expression of another important detoxifying enzyme, NQO1, in the spinal cords of animals with inflammatory or neuropathic pain. These results suggest that NQO1 does not play a relevant role in the mechanism of action of UFP-512 under chronic pain conditions.

The important role of the PI3K/Akt signaling pathway in the development of chronic pain is well accepted. Recent studies demonstrated the different participation of diverse PI3K isoforms in inflammatory and neuropathic pain (Pritchard et al., 2016; Liu et al., 2018). PI3K-β is involved in the nerve injury-induced sensitization of the spinal cord and contributes to neuropathic pain (Liu et al., 2018), but this β isoform does not participate in the development of inflammatory pain (Pritchard et al., 2016). In accordance, our findings demonstrated that sciatic nerve ligation activated the PI3Kβ/p-Akt signaling in spinal cords, but peripheral inflammation did not alter its expression. Notably, UFP-512 inhibited the sciatic nerve injury-induced upregulation of PI3K-β/p-Akt. The administration of specific inhibitors of PI3K-β isoform also reduces neuropathic pain (Liu et al., 2018). Therefore, the normalization of this pathway induced by UFP-512 suggests that the antiallodynic and antihyperalgesic effects of this DOR agonist during neuropathic pain partially occur via the inhibition of this nociceptive pathway.

The present study also evaluated the effect of UFP-512 on the expression of NOS2 during inflammatory and neuropathic pain. Peripheral inflammation or sciatic nerve injury did not modify the spinal cord protein levels of NOS2 at 14 or 28 days after CFA-injection or CCI-induction, respectively, which is consistent with a previous study (Redondo et al., 2017). The administration of UFP-512 did not alter NOS2 expression in animals with inflammatory or neuropathic pain, which indicates that this isoenzyme is not crucial for the effects of UFP-512.

The participation of MAPKs in the development and maintenance of chronic pain and the differential activation of MAPK pathways in neurons and glia (microglia and astrocytes) during inflammatory and neuropathic pain (Ji et al., 2009; Edelmayer et al., 2014) are well documented. Thus, under neuropathic pain conditions the expression of p-P38 is increased in microglia, and JNK is activated in astrocytes (Cao J. et al., 2015). Our findings support the role of MAPK in the maintenance of chronic pain because increased expression of p-JNK and p-ERK1/2 was observed in the spinal cords of animals with inflammatory or neuropathic pain. But curiously, while UFP-512 blocked the activation of these proteins in CFA-injected mice, this DOR agonist did not alter the elevated expression of p-JNK or p-ERK1/2 in the spinal cords of sciatic nerve-injured animals. Perhaps, repeated administration of this compound may be necessary to counteract the spinal activation of JNK and ERK1/2 induced by sciatic nerve injury. In summary, our results indicated that the alleviation of chronic inflammatory pain of UFP-512 was mainly produced via inhibition of JNK and ERK1/2 activation, and other mechanisms, such as the inhibition of PI3K/p-Akt, might be implicated in their antinociceptive effects under neuropathic pain conditions.

In addition, no changes in the expression of DOR were observed in the spinal cords of animals with inflammatory or neuropathic pain, which is consistent with a previous report (Obara et al., 2009). UFP-512 did not alter DOR expression during inflammatory or neuropathic pain.

The antidepressant-like activity of DOR agonists is well acknowledged. Indeed, DOR KO mice exhibit increased depressive-like behavior (Filliol et al., 2000), and several works demonstrated antidepressant-like effects produced by different DOR agonists in various behavioral paradigms (Lutz and Kieffer, 2013). In this lane, the antidepressant properties of UFP-512 in naïve animals were previously established in the forced swimming test (Aguila et al., 2007; Vergura et al., 2008). However and given that persistent neuropathic pain is generally associated with depressive-like behavior (Kawai et al., 2017), we evaluated the possible antidepressant activity of UFP-512 in animals with neuropathic pain. Our results confirmed the antidepressant-like effects of this drug after intraperitoneal administration in naïve animals (Aguila et al., 2007) and further demonstrated that UFP-512 inhibited the depressive-like behavior associated with persistent neuropathic pain (28 days after surgery). These data reveal that UFP-512 alleviates neuropathic pain and inhibits the depressive-like behavior associated with chronic pain.

## Conclusion

In conclusion, our study revealed the antinociceptive properties of UFP-512 in chronic inflammatory and neuropathic pain. Activation of the Nrf2-HO-1 pathway and the inhibition of p-JNK and p-ERK1/2 in the spinal cord may explain these effects in animals with inflammatory paint. Induction of the Nrf2-HO-1 pathway and blockade of PI3K/p-Akt signaling caused by nerve injury may be the principal reason for the antinociceptive effects of UFP-512 during neuropathic pain. Moreover, UFP-512 exhibited antidepressant properties in animals with depressive-like behavior associated with neuropathic pain, and its coadministration with SFN improved the antiallodynic effects of this DOR agonist in chronic pain. Consequently, this study suggests the administration of UFP-512 as a new alternative for the treatment of chronic inflammatory and neuropathic pain and the depressive-like behavior associated with neuropathic pain.

## Data Availability

All datasets generated for this study are included in the manuscript and/or the supplementary files.

## Author Contributions

SP performed the behavioral tests. SP, AD, NG, and SL performed the western blot assays. SP and OP performed the statistical analysis. GB contributed in new reagents or analytic tools. OP designed the study and wrote the manuscript. All authors contributed to manuscript revision, read, and approved the submitted version.

## Conflict of Interest Statement

The authors declare that the research was conducted in the absence of any commercial or financial relationships that could be construed as a potential conflict of interest.
